# A Sparse Reconstruction Approach for Identifying Gene Regulatory Networks Using Steady-State Experiment Data

**DOI:** 10.1371/journal.pone.0130979

**Published:** 2015-07-24

**Authors:** Wanhong Zhang, Tong Zhou

**Affiliations:** 1 School of Chemical Machinery, Qinghai University, Qinghai, China; 2 Department of Automation, Tsinghua University, Beijing, China; 3 Tsinghua National Laboratory for Information Science and Technology(TNList), Tsinghua University, Beijing, China; Leibniz-Institute for Farm Animal Biology (FBN), GERMANY

## Abstract

**Motivation:**

Identifying gene regulatory networks (GRNs) which consist of a large number of interacting units has become a problem of paramount importance in systems biology. Situations exist extensively in which causal interacting relationships among these units are required to be reconstructed from measured expression data and other a priori information. Though numerous classical methods have been developed to unravel the interactions of GRNs, these methods either have higher computing complexities or have lower estimation accuracies. Note that great similarities exist between identification of genes that directly regulate a specific gene and a sparse vector reconstruction, which often relates to the determination of the number, location and magnitude of nonzero entries of an unknown vector by solving an underdetermined system of linear equations *y* = Φ*x*. Based on these similarities, we propose a novel framework of sparse reconstruction to identify the structure of a GRN, so as to increase accuracy of causal regulation estimations, as well as to reduce their computational complexity.

**Results:**

In this paper, a sparse reconstruction framework is proposed on basis of steady-state experiment data to identify GRN structure. Different from traditional methods, this approach is adopted which is well suitable for a large-scale underdetermined problem in inferring a sparse vector. We investigate how to combine the noisy steady-state experiment data and a sparse reconstruction algorithm to identify causal relationships. Efficiency of this method is tested by an artificial linear network, a mitogen-activated protein kinase (MAPK) pathway network and the *in silico* networks of the DREAM challenges. The performance of the suggested approach is compared with two state-of-the-art algorithms, the widely adopted total least-squares (TLS) method and those available results on the DREAM project. Actual results show that, with a lower computational cost, the proposed method can significantly enhance estimation accuracy and greatly reduce false positive and negative errors. Furthermore, numerical calculations demonstrate that the proposed algorithm may have faster convergence speed and smaller fluctuation than other methods when either estimate error or estimate bias is considered.

## Introduction

In biological sciences, a significant task is to reconstruct GRNs from experiment data and other a priori information, which is a fundamental problem in understanding cellular functions and behaviors [1–3]. Spurred by advances in experimental technology, it is considerably interesting to develop a systematic method to provide new insights into the evolution of some target genes both in normal physiology and in human diseases. The present challenges in biological research are that the GRN is generally large-scaled and there are many restrictions on probing signals in biochemical experiments. These challenges make the problem of identifying a GRN much more difficult than other reverse engineering problems [4–6].

At present, numerous classical methods have been developed to unravel the interactions of GRNs, including Boolean network approaches [7, 8], Bayesian network inference [9, 10], partial or conditional correlation analysis [11, 12], differential equation analysis [13–15], and others. However, while their absolute and comparative performance remain poorly understood, some of results are associated with heavy computational burdens. Recently, an approach based on the total differential formula and total least-squares is proposed to infer a GRN from measured expression data [5, 16]. Although this method can weaken the effect of experimental uncertainty, there exist significant false positive and negative errors. To overcome these difficulties, researchers have obtained some positive and constructive results and improvements in inferring a GRN, including incorporating power law [17–19], distinguishing direct and indirect regulations [20], penalizing the regulation strength [21, 22], etc. However, these methods either have higher computing complexities or have lower estimation accuracies. Moreover, many methods may not be suited to large-scale network identifications. Then, how is it possible to accurately identify the causal relationships based on certain observable quantities extracted from partial measurements?

Note that great similarities exist between the network identification of a single gene (also called a node) and a sparse vector reconstruction, which often relates to the determination of the number, location and magnitude of the nonzero entries by solving the problem of underdetermined system of linear equations *y* = Φ*x*. Therefore, we propose a novel framework of sparse reconstruction to identify the structure of a GRN, so as to increase accuracy of causal regulation estimations, especially reduce their computational complexity.

In this paper, a linear description of the causal interacting relationships for a GRN is firstly established from steady-state experiment data based on nonlinear differential equations. Then, we adopt a sparse reconstruction algorithm to find the sparse solution of a large-scale underdetermined problem. Finally, some applications, on an artificially generated linear network with 100 nodes, a nonlinear MAPK signaling network with 103 proteins and the size 100 networks of the DREAM3 and DREAM4 challenges, are employed to demonstrate efficiency of this proposed algorithm. Moreover, we compare the performance of suggested approach with two state-of-the-art methods which are called subspace likelihood maximization (SubLM1 and SubLM2) methods [23], the widely adopted TLS method [24] and those available results on the DREAM project website. Computation results show that with a lower computational cost, the proposed method can significantly improve estimation accuracy and have competitive computational complexity. Overall, the main contributions of this paper can be stated as follows:

- Propose a general methodology to investigate the problem of GRN identification under the framework of sparse reconstruction, and validate that the sparse vector associated with the interaction among nodes can be accurately estimated based on a linearized model of the GRN.
- Adopt this approach to identify the underlying GRN without any knowledge about the topological features of underlying GRN, and demonstrate that this approach may have faster convergence speed and smaller fluctuation than other methods for a GRN inference.

## Materials and Methods

### A description of the GRN model

In a GRN with *n* genes, we assume that the dynamics of the *i*-th gene concentration *x*_*i*_ can be described by the following nonlinear differential equation:

$$\begin{array}{c} \frac{dx_{i}}{dt}=f\left(x_{1},x_{2},\cdots ,x_{n};\theta_{i}\right), \end{array}$$ (1)

in which *θ*_*i*_ stands for a kinetic parameter that can be changed through external perturbations. While each gene system in the GRN reaches an equilibrium, there exist *dx*_*i*_/*dt* = 0, *i* = 1, 2, ⋯, *n*, i.e. *f*(*x*_1_, *x*_2_, ⋯, *x*_*n*_;*θ*_*i*_) = 0. In order to quantitatively measure the direct effect among genes, we quantify the causal interaction between two genes in terms of the fractional changes Δ*x*_*i*_/*x*_*i*_ of the *i*-th gene caused by a change of another gene *j*. As argued in (Kholodenko et al., 2002) [25], at a stable equilibrium, the direct effect of the *j*-th gene on the *i*-th gene (*i* ≠ *j*) can be measured by *u*_*ij*_ which results in log-to-log derivatives:

$$\begin{array}{c} u_{ij}=\lim_{\Delta x_{i},\Delta x_{j}\to 0}\left(\frac{\Delta x_{i}/x_{i}}{\Delta x_{j}/x_{j}}\right)=\frac{\partial \;lnx_{i}}{\partial \;lnx_{j}}=-\frac{\partial f_{i}/\partial lnx_{j}}{\partial f_{i}/\partial lnx_{i}}. \end{array}$$ (2)

If *u*_*ij*_ = 0, it means that gene *j* has no causal effect on gene *i*. Whereas, if *u*_*ij*_ ≠ 0, it illustrates that there exist causal regulatory relationships. Then, according to above description, the gene *j* is regarded as the cause and the gene *i* the effect. That is, with the increase (decreases) of the concentration of gene *j*, the concentration of gene *i* also increases (decreases). Therefore, *u*_*ij*_ > 0 and *u*_*ij*_ < 0 represent activation and inhibition interaction respectively. Let $\Delta_{x_{j}}^{\left[s\right]}$ denote the variation of the steady state ${x_{j}}^{\left[s\right]}$ when a kinetic parameter changes by Δ_*θ*_*j*__. Then, taking the first-order Taylor expansions and normalization of each component at an equilibrium in the GRN, the following equation is obtained:

$$\begin{array}{c} \sum_{j=1}^{n}\frac{\partial f_{i}/\partial lnx_{j}}{\partial f_{i}/\partial lnx_{i}}\times \frac{\Delta_{x_{j}}^{\left[s\right]}}{x_{j}^{\left[s\right]}}\approx 0. \end{array}$$ (3)

Suppose that *m* experiments have been performed, and the relative variable quantity of the *j*-th gene in the ℓ-th experiment is denoted by $\phi_{j\ell }=\Delta_{x_{j}}^{\left[s\right]}/x_{j}^{\left[s\right]}$. Then, from the definition of *u*_*ij*_ and the above equation, we can easily obtain the causal relationship model of the *i*-th gene associated with the interaction among others as $\sum_{k=1,k\neq i}^{n}u_{ik}\phi_{k\ell }\approx \phi_{i\ell },\;\ell =1,2,\cdots ,m$. Moreover, while adjacency vector [*u*_*i*1_, ⋯*u*_*i*(*i* − 1)_, *u*_*i*(*i* + 1)_⋯*u*_*in*_]^T^ is denoted by *α*_*i*_, an *m* × (*n* − 1) measurement matrix Φ and the observation vector *b* ∈ *R* ^*m*^ are defined respectively as:

$$\begin{array}{c} \Phi =[\begin{array}{cccccc} \phi_{11} & \cdots & \phi_{i-1,1} & \phi_{i+1,1} & \cdots & \phi_{n1}\\ \phi_{12} & \cdots & \phi_{i-1,2} & \phi_{i+1,2} & \cdots & \phi_{n2}\\ \vdots & \cdots & \vdots & \vdots & \cdots & \vdots \\ \phi_{1m} & \cdots & \phi_{i-1,m} & \phi_{i+1,m} & \cdots & \phi_{nm} \end{array}], \end{array}$$

$$\begin{array}{c} b={\left[\phi_{i1},\phi_{i2},\cdots ,\phi_{im}\right]}^{T}, \end{array}$$

in which T denotes the operation of transposing. Then, the above causal regulation model can be compactly expressed as a linear equation:

$$\begin{array}{c} \Phi \alpha_{i}=b. \end{array}$$ (4)

The problem of inferring a GRN requires the precise estimation *α*_*i*_ using steady-state experiment data. In addition, the distribution of the degree of nodes in most GRNs obeys approximately the so-called power law as follows [26, 27]:

$$\begin{array}{c} P_{i}\left\{k\right\}=\{\begin{array}{cc} \mu k_{\min}^{-\gamma } & 1\leq k\leq k_{\min}\\ \mu k^{-\gamma } & k_{\min}\leq k\leq n \end{array}, \end{array}$$ (5)

where *k* denotes the number of nonzero entries of the sparse vector *α*_*i*_ and $\mu ={\left(k_{\text{min}}^{1-\gamma }+\sum_{k=\text{min}+1}^{n}k^{-\gamma }\right)}^{-1}$. That is, *k* is randomly generated using the power law distribution and the unknown vector *α*_*i*_ to be reconstructed is a sparse vector. Therefore, under the condition that both Φ and *b* are known, the purposes of this article are to reconstruct a sparse vector according to the above model. A distinctive characteristic of this problem to be identified is that both matrix Φ and vector *b* are corrupted by measurement noise. In the following section, the use of SmOMP for inferring GRN is described.

### A sparse reconstruction algorithm

The development of sparse reconstruction started at the seminal work in [28, 29]. These literatures elaborated that combining the ℓ_1_-minimization and random matrices can lead to efficient estimation of sparse vectors. Additionally, the researchers indicated that such notions have strong potential to be used in many applications. For an underdetermined system of linear equations:

$$\begin{array}{c} y=\Phi x, \end{array}$$ (6)

in which Φ ∈ R^*m*×*n*^ is called a measurement matrix. Note that *m* and *n* are at the same order of magnitude, or *m* is even much smaller than *n*. Thus, the above equations may have many solutions known from elementary linear algebra. However, we can seek a sparse solution with some a prior information on the signal sparsity and a certain matrix Φ. In sparse reconstruction, the aim is to find the sparse solution from the compressed measurement *y* and measurement matrix Φ. Then we have to add a constraint to the system so that we can limit the solution space. Specifically, we assume *x* is *k*-sparse, that is to say, the number *k* of nonzeros, called sparsity, is much less than *n*. So it can be obtained to solve the optimal solution of the ℓ_0_–*minimization* problem:

$$\begin{array}{ccc} \left(P_{0}\right) & \min_{x}{∥x∥}_{0} & s.t.\;\;y=\Phi x. \end{array}$$ (7)

As the present researches show, this is in fact a NP-hard problem. So it can be converted into solving the equivalent solution of the ℓ_1_–*minimization* problem:

$$\begin{array}{ccc} \left(P_{1}\right) & \min_{x}{∥x∥}_{1} & s.t.\;\;y=\Phi x. \end{array}$$ (8)

The classical algorithms find the solution of above sparse problem with minimal ℓ_1_ norm. Since these algorithms, based on convex optimization, can guarantee global optimum and have strong theoretical assurance, the problem can be solved via linear programming [30, 31]. However, the complexity is burdensome and unacceptable for the application of large-scale systems. Recently, greedy algorithms have received considerable attention as cost effective alternatives of the ℓ_1_–*minimization* [32, 33]. In the greedy algorithm family, stagewise orthogonal matching pursuit (StOMP) algorithm with the property either Φ that is random or that the nonzeros in *x* are randomly located, or both, is well suited to large-scale underdetermined applications in sparse vector estimations [34]. It can reduce computational complexity and has some attractive asymptotical statistical properties. However, the estimation speed is at the cost of accuracy violation. In this paper, an improvement algorithm on the StOMP which is called stagewise modified orthogonal matching pursuit (SmOMP), is suggested. This algorithm is more efficient at finding a sparse solution of large-scale underdetermined problems. Moreover, compared with StOMP, this modified algorithm can not only more accurately estimate parameters for the distribution of matched filter coefficients, but also improve estimation accuracy for the sparse vector itself [35].

SmOMP aims to estimate the distribution parameters for matched filter coefficients more accurately and improve the estimate accuracy of the sparse solution based on the true positive rate (TPR). Suppose that the undetermined linear system equation is *y* = Φ*x* in which *x* is the original sparse vector. SmOMP operates in *s* ≤ *S* stages, building up a sequence of approximations *x*_0_, *x*_1_, ⋯ by removing detected structure from a sequence of residual vectors *r*_0_, *r*_1_, ⋯. Starting from *x*_0_ = 0 and initial residue *r*_0_ = *y*, it iteratively constructs approximations by maintaining a sequence of estimates for the locations of the nonzeros in *x* as *I*_1_, …, *I*_*s*_.

At the *s*-th stage, we apply matched filtering to the current residual, obtaining a vector of residual correlations *c*_*s*_ = Φ^T^ *r*_*s*_. In StOMP, authors demonstrate that ⟨*ϕ*_*j*_, *r*_*s*_⟩, *j* = 1, 2, ⋯, *n*, are subject to the Gaussian distribution with zero or nonzero mean, which are corresponding to the null case (the first distribution) or the nonnull case (the second distribution):

- Null case: $\left\langle \phi_{j},r_{s}\right\rangle ∼\text{N}\left(0,\sigma_{s,1}^{2}\right),j\in I_{0}^{c}\cap I_{s-1}^{c}$;
- Nonnull case: $\left\langle \phi_{j},r_{s}\right\rangle ∼\text{N}\left(\mu_{s},\sigma_{s,2}^{2}\right),j\in I_{0}\cap I_{s-1}^{c}$;

in which *c* means the complement of a set.

We consider an *m*_*s*_-dimensional subspace, using *k*_*s*_ nonzeros out of *n*_*s*_ possible terms. Note that the coefficients of this subspace are obtained by matched filtering as follows:

$$\begin{array}{c} \langle \phi_{1},r_{s}\rangle ,\;\langle \phi_{2},r_{s}\rangle ,\ldots ,\;\langle \phi_{N_{s}},r_{s}\rangle . \end{array}$$ (9)

The above coefficients can be regarded as to be sampled from a mixture distribution and they are classified by hard threshold:

$$\begin{array}{c} J_{s}=\{j:|c_{s}(j)|>t_{s}\sigma_{s}\}. \end{array}$$ (10)

Since the first distribution can be approximately regarded as a Gaussian distribution with mean zero, the problem mentioned above is in essence a problem of hypothesis test. If the coefficients satisfy the above threshold condition, they are sampled from the second distribution, otherwise the first distribution. Therefore, we can estimate the variance of the first distribution iteratively by using the maximum likelihood method and the Wright criterion. In a nutshell, we adopt an outlier deletion method to estimate a more accurate variance of the first distribution, when the following condition of their relative error is satisfactory:

$$\begin{array}{c} |\sigma_{s^{\left(t+1\right)},1}-\sigma_{s^{\left(t\right)},1}|/\sigma_{s^{\left(t\right)},1}<\epsilon , \end{array}$$ (11)

here *σ*_*s*^(*t*)^,1_ stands for an estimate of the variance of the first distribution in the *t*-th iteration.

On the other hand, based on hard thresholding, we can yield a small set of large coordinates:

$$\begin{array}{c} {\tilde{J}}_{k_{s}}=\{j:|c_{s}(j)|>t_{s}\sigma_{s^{\left(t\right)},1}\}. \end{array}$$ (12)

For the somewhat interdependency of the columns in matrix Φ, some coefficients corresponding to the null case and the nonnull case may all be chosen into ${\tilde{J}}_{k_{s}}$. Therefore, we can refine ${\tilde{J}}_{k_{s}}$ so as to reduce the false positive rate (FPR) of this stage, by incorporating the cardinal number *k*_*s*_ of the support ${\tilde{J}}_{k_{s}}$ and TPR *β*_*s*_ computed from the nonnull distribution. Then, the maximum likelihood method is used to get the estimate of *μ*_*s*_, *σ*_*s*,2_. The calculation formula of *β*_*s*_ is

$$\begin{array}{c} \beta_{s}=Pr(|N(\mu_{s},\sigma_{s,2}^{2})|>t_{s}\sigma_{s,1}). \end{array}$$ (13)

We merge the subset of newly selected coordinates ${\tilde{J}}_{k_{s}}$ with the previous support estimate and project the vector *r*_*s*_ on space spanned by the columns of Φ belonging to the enlarged support ${\tilde{I}}_{s}$. We have

$$\begin{array}{c} {\tilde{x}}_{s}={\left(\Phi_{{\tilde{I}}_{s}}\right)}^{†}y={\left(\Phi_{{\tilde{I}}_{s}}^{T}\Phi_{{\tilde{I}}_{s}}\right)}^{-1}\Phi_{{\tilde{I}}_{s}}^{T}y, \end{array}$$ (14)

where † denotes the pseudo-inverse. According to the above result, we can derive the solution ${\tilde{x}}_{{\tilde{J}}_{k_{s}}}$ corresponding to ${\tilde{J}}_{k_{s}}$ for the *s*-th stage and sort the solution of this stage by size of amplitude. Then, select the refined suppose set *J*_*s*_ based on the *k*_*s*_ × *β*_*s*_. Finally, after updating support and solving a least-squares problem, a corresponding residual is produced. The SmOMP algorithm applies the next iteration as long as all the conditions of *s* < *S*, ‖*r*_*s*_‖ > *ϵ* and ${\tilde{J}}_{k_{s}}\neq \emptyset$ are satisfied.

In summary, on the basis of the whole algorithm framework, the procedure of SmOMP at every stage for reconstructing sparse vector consists of the following four main steps:

1. Compute the coefficients of this stage applying matched filtering and estimate the variance of the first distribution iteratively by using the outlier deletion method, according to Eq (10) and Eq (11).
2. Perform hard thresholding to find the significant supports and calculate the TPR *β*_*s*_ according to Eq (12) and Eq (13).
3. Update support set ${\tilde{I}}_{s}=I_{s-1}⋃{\tilde{J}}_{k_{s}}$ and get the approximation ${\tilde{x}}_{s}$ according to Eq (14), thereby obtain new support set *J*_*s*_ = {*j*_1_, *j*_2_, ⋯, *j*_⌊*k*_*s*_ × *β*_*s*_⌋_}, in which $|{\tilde{x}}_{j_{1}}|\geq |{\tilde{x}}_{j_{2}}|\geq \cdots \geq |{\tilde{x}}_{\left\lfloor k_{s}\times \beta_{s}\right\rfloor }|\geq \cdots$.
4. Have *x*_*s*_ = (Φ_*I*_*s*__)^†^ *y* by solving a least-squares problem and obtain the updated residual *r*_*s*_ = *y* − Φ*x*_*s*_.

The threshold parameter takes a value in the range *t*_*s*_ ∈ [2, 3]. It can also be chosen with false alarm control (FAC) or with false discover control (FDC). Since FAC strategy outperforms FDC strategy, we utilize FAC strategy in our simulation exclusively. For FAC strategy, *t*_*s*_ takes the value as the $\xi =(1-\frac{\alpha_{0}}{2})$ quantile of the standard normal distribution, where $\alpha_{0}=\frac{m-k}{S(n-k)}$. Additionally, in order to reduce the FPR of each stage of algorithm, the iteration number of the SmOMP may be much larger, but the iteration number will not surpass the sparsity *k* of vector *x*, which means that the computation complexity will not rise dramatically and thus the algorithm has a faster calculating speed.

From above relations of procedures, a theoretical condition is obtained to ensure that a sparse vector can be perfectly reconstructed by the SmOMP algorithm. A proof of this theorem is given in S1 Appendix.

**Theorem 1**. Let Λ denote the support of a sparse vector *x*_0_. Suppose that the final support set *I*_*s*_ of the estimation ${\hat{x}}_{s}$ contains indices not in Λ and Φ_*I*_*s*__ has full column rank. When the iteration loop of the SmOMP is finished, *x*_0_ can be perfectly recovered by the SmOMP. Then, we have: ${\hat{x}}_{s}=x_{0}$.

To illustrate that SmOMP is more efficient than StOMP in finding a sparse solution to underdetermined problems, we adopted the notion of the phase boundary suggested by Tanner and Donoho as a performance metric. This metric evaluates a specific parameter combination (*δ*, *ρ*) for successfully reconstructing a sparse vector, in which *δ* = *m*/*n* and *ρ* = *k*/*m*. The boundary of success phase calculated based on a large-system limit and the statistical behavior of matched coefficients is shown in Table 1.

**Table 1:** Comparison of the boundary of success phase at several values of indeterminacy *δ*.

*δ*	0.0500	0.2438	0.3602	0.5153	0.6122	0.7091	0.8061	1.0000
StOMP	0.1985	0.2955	0.3356	0.3813	0.4060	0.4289	0.4498	0.4879
SmOMP	0.2594	0.3794	0.4288	0.4898	0.5298	0.5716	0.6192	0.7982

From the above comparison, we can know that the boundary of success phase of SmOMP is higher than that of StOMP at several values of indeterminacy *δ*. Thus, given the number *m* of samples and the dimension *N* of sparse vector, according to *k* = *N* ⋅ *δ* ⋅ *ρ*, we can derive the maximum sparsity reconstructed successfully is about 0.7982*m* using SmOMP, but for StOMP, it is around 0.4879*m*. Of special note is that this is an issue of significant importance for potential application to large-scale systems. For example, it needs to reconstruct gene regulatory networks from the limited experiment data in systems biology. Although we are unsure about the sparsity of these networks, the underlying reverse-engineering problems may be solved by our algorithm as the maximum sparsity that can be successfully reconstructed by the algorithm is sufficiently large.

On the other hand, note that we discuss and analyze the computational complexities of the SmOMP algorithms. For a system of linear equations: *y* = Φ*x*, in which Φ ∈ R^*m*×*n*^ is called a measurement matrix, and *x* is denoted the causal adjacency vector of a node in the GRN with *n* nodes. At the *s*-th stage of SmOMP, the matched filtering is applied to the current residual, which is at cost of *mn* flops. Next, the step of hard thresholding requires at most 3*n* additional flops. A conjugate gradient solvers is exploited to get a new approximation *x*_*s*_, which involves at most 2*mn* + *O*(*n*) flops. The number of iterations of conjugate gradient is denoted as *τ* which is independent of *n* and *m*. Finally, a new residual is updated with additional *mn* flops. Therefore, SmOMP amounts to 2*S*(1 + *τ*)*mn*+3*Sn* + *O*(*n*) flops in the worst case, if the total number of SmOMP stages is denoted as *S*.

## Results and Discussion

A GRN is generally large-scaled and its structural property obeys approximately a power-law distribution. This insight gives us some important a prior information that a GRN may not be the sparsest network but must be a sparse network. Since the degrees of most nodes are very small, that a node has a high degree is in fact a low probability event or even a extremely low probability event in a GRN.

On the other hand, to sufficiently satisfy restricted isometry property (RIP) condition with a higher probability, we normalize measurement matrix Φ through dividing elements in each column by the ℓ_2_ norm of that column and corrupt it with Gaussian random noise.

In order to illustrate the effectiveness of the developed identification algorithms, tests are performed on an artificial linear network with 100 nodes, a MAPK pathway network with 103 proteins and the size 100 network of the DREAM3 and DREAM4 challenges. Moreover, we compare the proposed approach with the algorithms of StOMP, SubLM1, SubLM2, TLS and those available results on the DREAM project.

### Assessment metrics

The performance evaluation of GRN is different from that of traditional estimation problems, and the main evaluation metrics are based on medical diagnosis evaluation system. For a GRN consisting of *n* nodes, we consider that the actual direct effect of the *j*-th node on the *i*-th node is denoted as *x*_*ij*_ and its estimate ${\hat{x}}_{ij}$, *i*, *j* = 1, 2, ⋯, *n*. Moreover, the total number of *x*_*ij*_ = 0 and *x*_*ij*_ ≠ 0 is represented by N and P respectively. Furthermore, let TP, FP FS TN and FN denote the number of true positive, false positive, false sign, true negative and false negative respectively. Then we can define the assessment metrics as follows:

- FP rate (FPR, also called misdiagnostic rate):
$\frac{\text{FP}}{\text{N}}=\text{1—}\;\frac{\text{TN}}{\text{N}}=\frac{\#(x_{ij}=0\;\text{but}\;{\hat{x}}_{ij}\neq 0)}{\#(x_{ij}=0)}$.
- TP rate (TPR, also called sensitivity or recall):
$\frac{\text{TP}}{\text{P}}=\text{1—}\;\frac{\text{FN}}{\text{P}}=\frac{\#(x_{ij}\neq 0\;\text{and}\;{\hat{x}}_{ij}\neq 0)}{\#(x_{ij}\neq 0)}$.
- FN rate (FNR, also called missed diagnosis rate):
$\frac{\text{FN}}{\text{P}}=\text{1—}\;\frac{\text{TP}}{\text{P}}=\frac{\#(x_{ij}\neq 0\;\text{and}\;{\hat{x}}_{ij}=0)}{\#(x_{ij}\neq 0)}$.
- TN rate (TNR, also called specificity):
$\frac{\text{TN}}{\text{N}}=\text{1—}\;\frac{\text{FP}}{\text{N}}=\frac{\#(x_{ij}=0\;\text{and}\;{\hat{x}}_{ij}=0)}{\#(x_{ij}=0)}$.
- Positive predictive value (PPV, also called true discovery rate or precision):
$\frac{\text{TP}}{\text{TP + FP}}=\frac{\#(x_{ij}\neq 0\;\text{and}\;{\hat{x}}_{ij}\neq 0)}{\#(x_{ij}\neq 0\;\text{and}\;{\hat{x}}_{ij}\neq 0)+\#(x_{ij}=0\;\text{but}\;{\hat{x}}_{ij}\neq 0)}$.

Of special note is that some typically adopted metrics are used to evaluate our algorithm performance in GRN identifications, such as receiver operating characteristics (ROC) curve, precision recall (PR) curve, area under a ROC curve (AUROC), area under a PR curve (AUPR), and so on. The ROC curve and PR curve are traced by scanning all possible decision boundaries. To be more specific, the ROC curve graphically explores the tradeoff between the complementary TPR and FPR as the threshold value is varied. If the points of ROC curve are closer to the upper-left-hand corner, the sensitivity and specificity are more valid. Similarly, the PR curve graphically explores the tradeoff between the precision and recall. Note that although both ROC and PR curves are commonly used to evaluate network predictions, given the assumption that the network is sparse PR curves are to be preferred (class imbalance: many more negatives than positives) [36]. Intuitively, PR better assesses correctness of predictions at the top of the list, which is what matters most for biological applications. That is, compared with the ROC curve, the PR curve can testify whether the first few predictions at the top of the prediction list are correct. This implies that the higher these points of the upper-left-hand corner are, the more reliable the estimation performances. Furthermore, the AUROC and the AUPR represent a single number that summarizes the ROC and PR tradeoff respectively. Clearly, the larger the values of these metrics are, the higher accuracy the prediction.

### An artificial linear network

In this application, we use a linear model *A*_0_ *X*_0_ = *B*_0_ to describe the GRN, where *A*_0_ ∈ *R* ^*m*×*n*^ is a measurement matrix whose entries are independently and uniformly sampled from [1, 10], *X*_0_ ∈ *R* ^*n*×*n*^ denotes the causal adjacency matrix of the GRN with *n* = 100 nodes. In this numerical simulation, every column of *X*_0_ is independently generated according to the next three steps.

- For each column of *X*_0_, the number *k* of nonzero entries is randomly generated using the power law distribution. Note that the parameters of power law take the empirical values as *k*_*min*_ = 1 and *γ* = 2.5.
- Locations of non-zero elements are determined by the function of randperm in MATLAB for random permutations. That is, elements of the set {1, 2, …, 100} are at first randomly permuted, and then the first k elements are adopted as the locations of the rows in this column with non-zero entries. Denote them by ${\ell_{\alpha }|}_{\alpha =1}^{k}$.
- The entry of the ℓ_*α*_-th row of this column is generated independently according to a uniform distribution over [−2, −*ρ*_*a*_]⋃[*ρ*_*a*_, 2], *α* = 1, 2, ⋯, *k*. Here, *ρ*_*a*_ = 10^−5^ represents an acceptable magnitude bound. All the other entries are assigned to be zero.

Then, matrix *A* = *A*_0_ + *ω*_*A*_ and *B* = *A*_0_ *X*_0_ + *ω*_*B*_ are generated, where *ω*_*A*_ and *ω*_*B*_ are are drawn from a normal distribution N(0, *σ* ^2^). After the production of matrices A and B, every column of *X*_0_ is estimated on the basis A and B.

**Fig 1:**
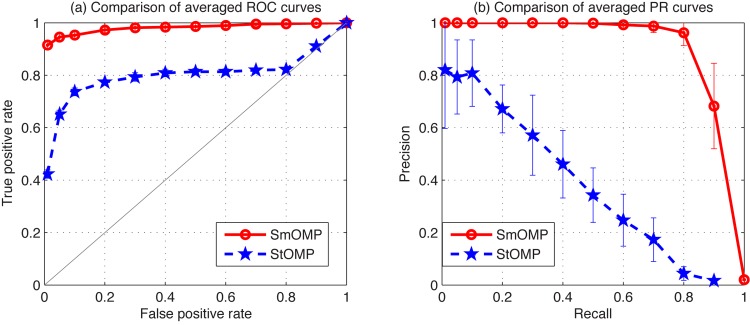
Reconstruction performance of the StOMP and SmOMP algorithms with *m* = 80, *σ* = 0.3 for the artificial network inference. (a) Comparison of averaged ROC curves. (b) Comparison of averaged PR curves.

We at first compare our algorithm with the StOMP onto this model when the measurement dimensions *m* = 80. The parameter of FAC *α*_0_ = 0.3 and the empirical standard deviation *σ* = 0.1. Moreover, 500 independent simulation trails have been performed to investigate the statistical properties of estimates. Averaged ROC and PR curves of this example are shown in Fig 1, respectively. From performance results, we can see that the reconstruction performance of SmOMP is significantly better than that of StOMP.

**Fig 2:**
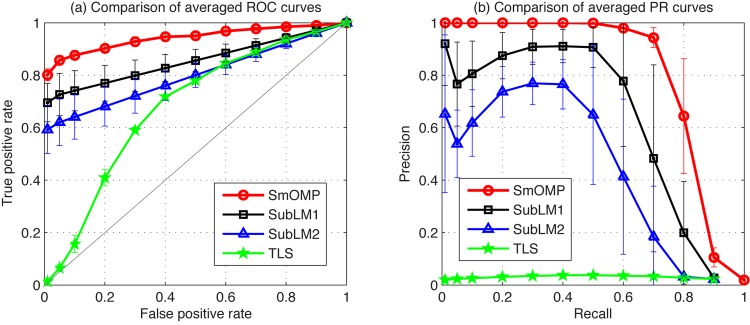
Reconstruction performance of the SmOMP, SubLM1, SubLM2 and TLS algorithms with *m* = 1000, *σ* = 2.0 for the artificial network inference. (a) Comparison of averaged ROC curves. (b) Comparison of averaged PR curves.

On the other hand, we consider two novel algorithms, which are also called SubLM1 and SubLM2 proposed by Zhou et al.(2010). These methods incorporate angle minimization of subspaces and likelihood maximization to infer causal regulation. We compare the SmOMP with the SubLM1, SubLM2 and TLS algorithms using this linear system. The simulation results of the corresponding ROC and PR curves are shown in Fig 2 at *m* = 1000 under the noise level *σ* = 2.0. Corresponding mean values and standard deviations (std) of AUROC and AUPR, and the averaged runtime of each trail are tabulated in Table 2.

**Table 2:** Estimation performances for the artificial linear network.

*Metrics*	AUROC/AUPR (*mean*±*std*) × 10^−2^, Runtime (*second*)			
*Methods*	SmOMP	SubLM1	SubLM2	TLS
AUROC	92.3±3.40	84.6±4.18	79.8±3.93	68.6±2.91
AUPR	76.2±4.55	60.4±9.64	43.4±8.77	3.24±0.13
Runtime	6.3248	7.1775	3.8437	7.6085

It is obvious that the proposed method has distinguished advantages over SubLM1, SubLM2 and TLS algorithm in parametric estimation accuracy, FPR and TPR. In addition, when entries of *A*_0_ take independent and uniform random samples from [−10, −1] ∪ [1, 10], the suggested method always outperforms the others.

### A nonlinear MAPK pathway network

This MAPK pathway model, it consists of 103 chemical elements and is described by a set of first-order ordinary nonlinear differential equations which take completely the same form as Eq (1). This model is originally built in Schoeberl et al.(2002) and capable of explaining many biological observations. Readers interested in details of this differential equations, their parameters as well as model structure, are recommended to refer to the original paper. In this simulation, 37 species whose approximation errors are relatively small are chosen to test the performance of algorithms. To generate the data using numerical simulation, experimental designs and parameter settings are given as follows:

- The Jacobian matrix of the nonlinear function vector $f_{i}{({x_{j}|}_{j=1}^{103},{\theta_{k}|}_{k=1}^{247})|}_{i=1}^{103}$ is at first computed at the selected stable equilibrium *x* ^[*s*]^, which is further used to calculate the actual interactions among chemical elements. That is, the real causal interaction value is computed according to the following formula:
  $$\begin{array}{c} u_{ij}=\frac{\partial \ln x_{i}}{\partial \ln x_{j}}{|}_{x=x^{\left[s\right]}}=\left(-\frac{\partial f_{i}}{\partial \ln x_{j}}/\frac{\partial f_{i}}{\partial \ln x_{i}}\right){|}_{x=x^{\left[s\right]}}. \end{array}$$
- To apply the suggested algorithms, the parameters of Eq (5) for the power law are required. Based on above results, parameters of the power law are estimated through counting the number of nonzero *u*_*ij*_ with a fixed *i*, *i*, *j* = 1, 2, ⋯, 103; and fitting the logarithm of the corresponding empirical probabilities. Using this method, $\hat{\gamma }=0.8000$, and ${\hat{k}}_{min}=1$ are obtained.

In data generations, kinetic parameters $\theta_{k}{|}_{k=1}^{247}$ and initial values of $x_{j}{|}_{j=1}^{103}$ are changed in a way similar to that of Andrec et al. (2005) and Kholodenko et al. (2002). That is, when direct influences on the *i*-th species are to be estimated, only the values of these *θ*_*k*_, *k* ∈ 1, 2, ⋯, 247, are permitted to be changed or perturbed which do not explicitly alter the value of the nonlinear function *f*_*i*_(*x*, *p*). More specifically, an appropriate *θ*_*k*_ is selected together with 8 ∼ 12 *x*_*k*_s that are respectively changed to 0.9999*α*_*j*_ *p*_*j*_ for all the simulated time and 0.9999*β*_*k*_ *x*_*k*_ at the initial time. Here, both *α*_*j*_ and *β*_*j*_ are independent and uniform random samples from [0.9, 1]. Steady-state concentration of every species in the network is calculated before and after a perturbation using the toolbox *Simulink* of the commercial software MATLAB. To every calculated relative concentration change at the steady states, that is $\Delta_{x_{j}}^{\left[s\right]}/x_{j}^{\left[s\right]}$, a random number is added which is independently generated according to the normal distribution with zero mean and standard deviation 10^−5^. Perturbation experiments are performed totally *m* = 145 times. Thus experimental data matrix *A* of the *i*-th species is obtained. Then,

$$\begin{array}{c} \Phi =A\left(:,\left[1:\left(i-1\right),\left(i+1\right):103\right]\right),\;b_{i}=A\left(:,i\right). \end{array}$$

**Fig 3:**
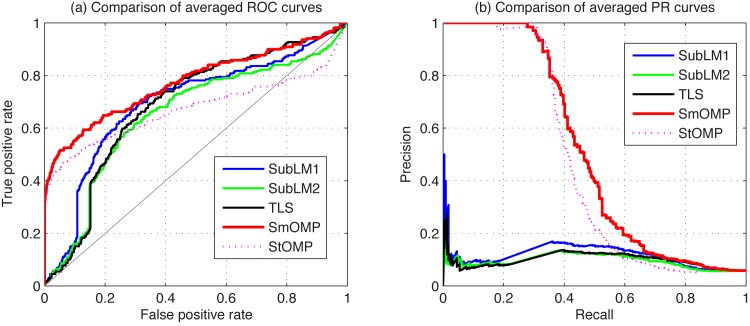
Comparison of the averaged ROC and PR curves in the MAPK network identification using the SubLM1, SubLM2, TLS, SmOMP and StOMP algorithms. (a) Averaged ROC curves. (b) Averaged PR curves.

We consider five algorithms for comparison in a nonlinear MAPK network, which are SubLM1, SubLM2, TLS, SmOMP and StOMP. The averaged ROC and PR curves are shown in Fig 3. Additionally, the performance metrics of AUROC and AUPR and the averaged runtime are shown in Table 3. From these results, it is obvious that the SmOMP algorithm outperforms other methods.

**Table 3:** Reconstruction performance and the averaged runtime for a nonlinear MAPK network.

	AUROC/AUPR (*mean*±*std*) × 10^−2^, Runtime (*second*)					
*Metrics*	SmOMP	StOMP	SubLM1	SubLM2	TLS
AUROC	82.32±2.40	78.12±2.33	77.33±3.68	75.15±3.15	77.82±3.46
AUPR	53.21±3.79	50.11±3.99	13.02±3.94	12.44±3.21	9.24±1.62
Runtime	2.4841	2.4320	10.6050	9.0410	1.2294

On the other hand, convergence properties of the proposed method are investigated by some numerical simulations. In these investigation, we selected the (EGF-EGFRI)2 protein which is the 11th node of this MAPK pathway network, to identify the causal interactions from other proteins with data length increment. In every simulation trail, 500 equally distributed samples are taken from interval [20, 10000] for the data length. At a fixed data length, we calculate the mean square of the estimate errors and squares of estimate bias which are defined respectively as follow:

$$\begin{array}{c} \frac{1}{M}\sum_{h=1}^{M}{\left({\hat{x}}^{\left[h\right]}-x\right)}^{T}\left({\hat{x}}^{\left[h\right]}-x\right), \end{array}$$ (15)

$$\begin{array}{c} {\left(\frac{1}{M}\sum_{h=1}^{M}{\hat{x}}^{\left[h\right]}-x\right)}^{T}\left(\frac{1}{M}\sum_{h=1}^{M}{\hat{x}}^{\left[h\right]}-x\right). \end{array}$$ (16)

Here, ${\hat{x}}^{\left[h\right]}$ represents the estimate for the actual regulation coefficient vector *x* in the *h*-th estimation of *M* experiments. To compute the ensemble average estimation error and estimation bias at every data length, 100 simulation are performed for each set of numerical experiment settings. From calculated results of these two specifications respectively, we can know that the proposed method may have faster convergence speed and smaller stochastic fluctuation for the estimate errors or the estimation bias than other algorithms. Meanwhile, these results show the sparse reconstruction algorithm is not only suitable for some high-dimensional data, but also for linear lower-dimension problem. Therefore, the identification performance of the SmOMP to reconstruct the causal relationship of the GRN is significantly better than the other algorithms. Of special note is that the processing time of SmOMP is much less than that of the SubLM1, SubLM2 and TLS which can be clearly observed from the runtime comparison.

### Application to the DREAM networks

DREAM is an international initiative with the aim of evaluating methods for biomolecular network identification in an unbiased way [37–40]. To evaluate the proposed algorithm, it has also been applied to the *in* *silico* steady state datasets of the size 100 networks of the DREAM3 and DREAM4 challenges. Each challenge consists five different benchmark networks with 100 genes which are obtained through extracting some important and typical modules from actual biological networks. In these challenges, the participants had to predict the topologies of five 100-gene networks, and were provided with steady state gene expression levels from wild-type, knockout data. The wild-type file contained 100 steady-state levels of the unperturbed network. The knockout data consisted of 100 rows of steady-state values, and each row is obtained after deleting one of the 100 genes. More detailed explanations can be found on the website of the DREAM project at http://wiki.c2b2.columbia.edu/dream/. Predictions are compared with the actual structure of the networks by the DREAM project organizers using the AUROC and the AUPR metrics in topology prediction accuracy evaluations. Then, we can compute *p*(*AUROC*) and *p*(*AUPR*), which are the probability that a given or larger area under the curve value is obtained by random ordering of the potential network links. Distributions for AUROC and AUPR were estimated from 100,000 instances of random network link permutations. Based on these *p*-values, a final score in each subchallenge is calculated as follows:

$$\begin{array}{c} Score=-\frac{1}{2}\log_{10}[{\left(\prod_{i=1}^{5}p_{i}(AUROC)\right)}^{\frac{1}{5}}\times {\left(\prod_{i=1}^{5}p_{i}(AUPR)\right)}^{\frac{1}{5}}]. \end{array}$$ (17)

Note that a larger score indicates a greater statistical significance of the adopted reconstruction algorithm for the network prediction.

**Fig 4:**
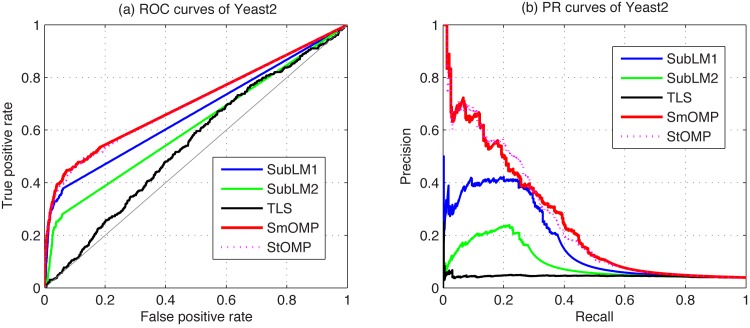
Comparison of the ROC and PR curves in the DREAM3 identification using the SubLM1, SubLM2, TLS, SmOMP and StOMP algorithms. (a) ROC curves of Yeast2. (b) PR curves of Yeast2.

**Fig 5:**
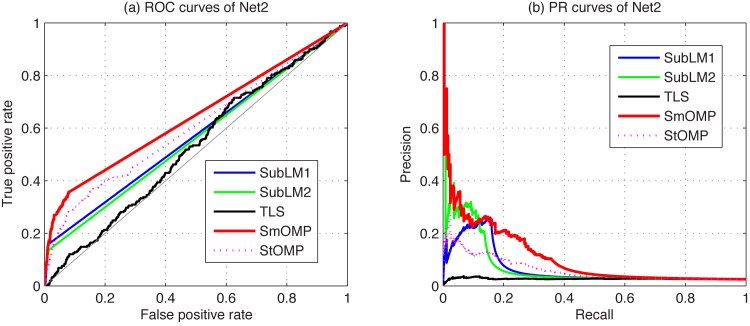
Comparison of the ROC and PR curves in the DREAM4 identification using the SubLM1, SubLM2, TLS, SmOMP and StOMP algorithms. (a) ROC curves of Net2. (b) PR curves of Net2.

We compare the SmOMP with the StOMP, SubLM1, SubLM2 and TLS algorithms for the DREAM3 and DREAM4 using only steady-state data. The corresponding ROC and PR curves of some typical estimations are respectively shown in Fig 4 for the Yeast2 in DREAM3, and Fig 5 for the Net2 in DREAM4. From these figures, it is obvious that the SmOMP algorithm is best among these five methods. Moreover, for every network of the DREAM3 and DREAM4 challenges, reconstruction results are respectively presented in Table 4. From these results and those available on the DREAM project website, we can conclude that the final score of proposed algorithm is much higher than Teams 296 which is top scorer among 22 participated teams in the DREAM3 challenge, and the estimation performances of the SmOMP algorithm significantly outperform Teams 236 which has been ranked the 14th place among 19 participated teams in the DREAM4 challenge. In addition, since our estimation procedures have significantly lower computational complexities, the SmOMP algorithm may be well appropriate and competent to identify large-scale GRNs. To be more specific, for the best of these challenges in DREAM3, it reported that 78h have been consumed to obtain an estimate a high-end cluster. However, utilizing a personal computer which is equipped with a 2.2 GHz CPU processor and a 2.0 GB RAM, SmOMP is required the averaged runtime 0.2730s, 0.5604s and 1.0538s for the 10-node, 50-node and 100-node network of the DREAM3 Ecoli1, respectively.

**Table 4:** Reconstruction performance for the DREAM3 and DREAM4 in the size 100 subchallenges.

	Metrics	SmOMP	StOMP	SubLM1	SubLM2	TLS	Top scorer
DREAM3	Score	49.7099	48.9620	32.0813	14.1991	2.7557	45.4430
	Runtime	1.3316s	1.0420s	1.1522s	0.1002s	1.4134s	78h
DREAM4	Score	15.9873	11.402	9.4248	5.8975	0.8830	71.5890
	Runtime	0.9620s	0.8204s	1.3696s	0.6042s	1.2569s	– – –

**Fig 6:**
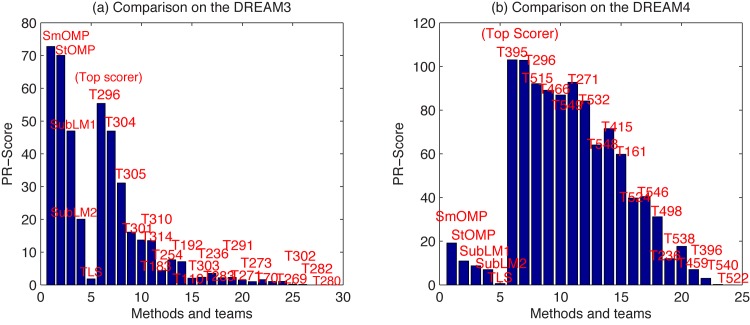
PR-Score of the SubLM1, SubLM2, TLS, SmOMP and StOMP algorithms and all teams from the DREAM project. (a) Comparison on the DREAM3. (b) Comparison on the DREAM4.

On the other hand, we compare all the teams available in DREAM3 and DREAM4 challenges and the methods applied in this paper based on the score of the AUPR only (Eq (17) without the AUROC term, and called as PR-Score). A figure about this PR-Score for them as bar plot is shown in Fig 6. Note that the scores of all teams included here are obtained directly from the website of the DREAM project.

From these results, we can see that the PR-score of SmOMP is the best among all teams and other methods for the DREAM3 challenge. However, in the DREAM4 challenge, performance of SmOMP is very poor. This may possibly be due to that the adopted assumption has been seriously deteriorated that measurement noises are independently subject to the Gaussian distribution. In addition, unlike ordinary differential equations for DREAM3, the training data in DREAM4 are generated based on stochastic differential equations to model internal noise in the dynamics of networks.

## Concluding Remarks

A sparse reconstruction approach is proposed in this paper to identify the causal relationship of a GRN from steady-state experiment data. We at first introduce a linearized method to model the causal relationship for a large-scale GRN based on nonlinear differential equations. Then, we investigate application of a sparse reconstruction algorithm to solve sparse problems of lager-scale underdetermined system. Besides, we demonstrate efficiency of this approach through identifying the causal relationships of an artificial linear network, a MAPK network and some *in* *silico* networks of DREAM challenges. Finally, we compare the performance of the suggested approach with two state-of-the-art algorithms, a widely adopted TLS method and those available results on the DREAM project website. Actual computations with noisy steady-state experiment data show that with a lower computational cost, the proposed method has significant advantages on estimation accuracy and has a much faster convergence speed.

It is worthwhile to mention that while most of the reported results are encouraging, this method is still far from satisfaction of practical application requirements. This has been made very clear by the unsatisfactory performances with the challenge of DREAM4. Inspired by these results, there are two further researches for the causal relationship of the large-scale GRNs. On one hand, we are interested in investigating the overall topology identification by incorporating the power law distribution of the GRNs. On the other hand, using this sparse reconstruction approach to corroborate the actual gene networks obtained by biological experiments is part of our future work.

## Supporting Information

S1 Appendix Proof of Theorem 1. (PDF)

## References

[1] Hecker M, Lambeck S, Toepfer S, Van Someren E, Guthke R (2009) Gene regulatory network inference: data integration in dynamic modelsa review. Biosystems 96: 86–103. 10.1016/j.biosystems.2008.12.004 19150482

[2] Feala JD, Cortes J, Duxbury PM, Piermarocchi C, McCulloch AD, et al (2010) Systems approaches and algorithms for discovery of combinatorial therapies. Wiley Interdisciplinary Reviews: Systems Biology and Medicine 2: 181–193. 20836021 10.1002/wsbm.51

[3] Barabasi AL, Oltvai ZN (2004) Network biology: understanding the cells functional organization. Nature Reviews Genetics 5: 101–113. 10.1038/nrg1272 14735121

[4] Akutsu T, Kuhara S, Maruyama O, Miyano S (2003) Identification of genetic networks by strategic gene disruptions and gene overexpressions under a boolean model. Theoretical Computer Science 298: 235–251. 10.1016/S0304-3975(02)00425-5

[5] Andrec M, Kholodenko BN, Levy RM, Sontag E (2005) Inference of signaling and gene regulatory networks by steady-state perturbation experiments: structure and accuracy. Journal of theoretical biology 232: 427–441. 10.1016/j.jtbi.2004.08.022 15572066

[6] Gardner TS, Di Bernardo D, Lorenz D, Collins JJ (2003) Inferring genetic networks and identifying compound mode of action via expression profiling. Science 301: 102–105. 10.1126/science.1081900 12843395

[7] Shmulevich I, Dougherty ER (2010) Probabilistic Boolean networks: the modeling and control of gene regulatory networks. siam.

[8] Yun Z, Keong KC (2004) Reconstructing boolean networks from noisy gene expression data. In: Control, Automation, Robotics and Vision Conference, 2004. ICARCV 2004 8th. IEEE, volume 2, pp. 1049–1054.

[9] Ferrazzi F, Sebastiani P, Ramoni MF, Bellazzi R (2007) Bayesian approaches to reverse engineer cellular systems: a simulation study on nonlinear gaussian networks. BMC bioinformatics 8: S2 10.1186/1471-2105-8-S5-S2 17570861 PMC1892090

[10] Li Z, Li P, Krishnan A, Liu J (2011) Large-scale dynamic gene regulatory network inference combining differential equation models with local dynamic bayesian network analysis. Bioinformatics 27: 2686–2691. 10.1093/bioinformatics/btr454 21816876

[11] Penfold CA, Buchanan-Wollaston V, Denby KJ, Wild DL (2012) Nonparametric bayesian inference for perturbed and orthologous gene regulatory networks. Bioinformatics 28: i233–i241. 10.1093/bioinformatics/bts222 22689766 PMC3371854

[12] Rice JJ, Tu Y, Stolovitzky G (2005) Reconstructing biological networks using conditional correlation analysis. Bioinformatics 21: 765–773. 10.1093/bioinformatics/bti064 15486043

[13] Karlebach G, Shamir R (2008) Modelling and analysis of gene regulatory networks. Nature Reviews Molecular Cell Biology 9: 770–780. 10.1038/nrm2503 18797474

[14] Liu B, de La Fuente A, Hoeschele I (2008) Gene network inference via structural equation modeling in genetical genomics experiments. Genetics 178: 1763–1776. 10.1534/genetics.107.080069 18245846 PMC2278111

[15] Iba H (2008) Inference of differential equation models by genetic programming. Information Sciences 178: 4453–4468. 10.1016/j.ins.2008.07.029

[16] Sontag E (2008) Network reconstruction based on steady-state data. Essays Biochem 45: 161–176. 10.1042/BSE0450161 18793131

[17] Albert R (2005) Scale-free networks in cell biology. Journal of cell science 118: 4947–4957. 10.1242/jcs.02714 16254242

[18] Vidal M, Cusick ME, Barabasi AL (2011) Interactome networks and human disease. Cell 144: 986–998. 10.1016/j.cell.2011.02.016 21414488 PMC3102045

[19] Xiong J, Zhou T (2012) Gene regulatory network inference from multifactorial perturbation data using both regression and correlation analyses. PloS one 7: e43819 10.1371/journal.pone.0043819 23028471 PMC3448649

[20] Wang Yl, Zhou T (2012) A relative variation-based method to unraveling gene regulatory networks. PloS one 7: e31194 10.1371/journal.pone.0031194 22363578 PMC3282721

[21] Chang R, Stetter M, Brauer W (2008) Quantitative inference by qualitative semantic knowledge mining with bayesian model averaging. Knowledge and Data Engineering, IEEE Transactions on 20: 1587–1600. 10.1109/TKDE.2008.89

[22] Xiong J, Zhou T (2013) Parameter identification for nonlinear state-space models of a biological network via linearization and robust state estimation. In: Control Conference (CCC), 2013 32nd Chinese. IEEE, pp. 8235–8240.

[23] Zhou T, Wang YL (2010) Causal relationship inference for a large-scale cellular network. Bioinformatics 26: 2020–2028. 10.1093/bioinformatics/btq325 20554691

[24] Berman P, DasGupta B, Sontag E (2007) Randomized approximation algorithms for set multicover problems with applications to reverse engineering of protein and gene networks. Discrete Applied Mathematics 155: 733–749. 10.1016/j.dam.2004.11.009

[25] Kholodenko BN, Kiyatkin A, Bruggeman FJ, Sontag E, Westerhoff HV, et al (2002) Untangling the wires: a strategy to trace functional interactions in signaling and gene networks. Proceedings of the National Academy of Sciences 99: 12841–12846. 10.1073/pnas.192442699 PMC130547 12242336

[26] Clauset A, Shalizi CR, Newman ME (2009) Power-law distributions in empirical data. SIAM review 51: 661–703. 10.1137/070710111

[27] Zhou T, Xiong J, Wang YL (2012) GRN topology identification using likelihood maximization and relative expression level variations. In: Control Conference (CCC), 2012 31st Chinese. IEEE, pp. 7408–7414.

[28] Candes EJ, Tao T (2006) Near-optimal signal recovery from random projections: Universal encoding strategies? Information Theory, IEEE Transactions on 52: 5406–5425. 10.1109/TIT.2006.885507

[29] Donoho DL (2006) Compressed sensing. Information Theory, IEEE Transactions on 52: 1289–1306. 10.1109/TIT.2006.871582

[30] Sarvotham S, Baron D, Baraniuk RG (2006) Compressed sensing reconstruction via belief propagation. preprint.

[31] Candes EJ (2008) The restricted isometry property and its implications for compressed sensing. Comptes Rendus Mathematique 346: 589–592. 10.1016/j.crma.2008.03.014

[32] Wang J, Kwon S, Shim B (2012) Generalized orthogonal matching pursuit. Signal Processing, IEEE Transactions on 60: 6202–6216. 10.1109/TSP.2012.2218810

[33] Needell D, Vershynin R (2009) Uniform uncertainty principle and signal recovery via regularized orthogonal matching pursuit. Foundations of computational mathematics 9: 317–334. 10.1007/s10208-008-9031-3

[34] Donoho DL, Tsaig Y, Drori I, Starck JL (2012) Sparse solution of underdetermined systems of linear equations by stagewise orthogonal matching pursuit. Information Theory, IEEE Transactions on 58: 1094–1121. 10.1109/TIT.2011.2173241

[35] Zhang WH, Huang Bx, Zhou T (2013) An improvement on stomp for sparse solution of linear underdetermined problems. In: Control Conference (CCC), 2013 32nd Chinese. IEEE, pp. 1951– 1956.

[36] Davis J, Goadrich M (2006) The relationship between precision-recall and roc curves. In: Proceedings of the 23rd international conference on Machine learning. ACM, pp. 233–240.

[37] Pinna A, Soranzo N, De La Fuente A (2010) From knockouts to networks: establishing direct cause-effect relationships through graph analysis. PloS one 5: e12912 10.1371/journal.pone.0012912 20949005 PMC2952592

[38] Prill RJ, Marbach D, Saez-Rodriguez J, Sorger PK, Alexopoulos LG, et al (2010) Towards a rigorous assessment of systems biology models: the dream3 challenges. PloS one 5: e9202 10.1371/journal.pone.0009202 20186320 PMC2826397

[39] Marbach D, Schaffter T, Mattiussi C, Floreano D (2009) Generating realistic in silico gene networks for performance assessment of reverse engineering methods. Journal of computational biology 16: 229–239. 10.1089/cmb.2008.09TT 19183003

[40] Marbach D, Prill RJ, Schaffter T, Mattiussi C, Floreano D, et al (2010) Revealing strengths and weaknesses of methods for gene network inference. Proceedings of the National Academy of Sciences 107: 6286–6291. 10.1073/pnas.0913357107 PMC2851985 20308593

