# Supplementary material for: A Sparse Reconstruction Approach for Identifying Gene Regulatory Networks Using Steady-State Experiment Data
Source: PLoS One. 2015 Jul 24;10(7):e0130979. doi: 10.1371/journal.pone.0130979 (PMC4514654; doi:10.1371/journal.pone.0130979)
Supplement: S1 Appendix — (PDF) [file pone.0130979.s001.pdf]

S1 Appendix: Proof of Theorem 1

Let $\Omega = \{1, 2, \cdots, n\}$, then $\Lambda = \{i | i \in \Omega, x_i \neq 0\}$ represents the support of vector x_0. For $I_s \subseteq \Omega$, $|I_s|$ is the cardinality of I_s. $\Phi_{I_s} \in \mathrm{R}^{m \times |I_s|}$ is a submatrix of $\Phi \in \mathrm{R}^{m \times n}$ that only contains columns indexed by $|I_s|$. $I_s - \Lambda$ means the set of all elements contained in I_s but not in Λ.

Since Φ_{I_s} is full column rank, then $\Phi^{\dagger}_{I_s} = (\Phi^T_{I_s} \Phi_{I_s})^{-1} \Phi^T_{I_s}$ is the pseudoinverse Φ_{I_s} and $\mathrm{P}^{\perp}_{I_s} = \mathrm{I} - \mathrm{P}_{I_s}$, in which $\mathrm{P}_{I_s} = \Phi_{I_s} \Phi^{\dagger}_{I_s}$ is the projection onto the span of columns in Φ_{I_s}. Meanwhile, it is worthwhile to mention that the residual r_s is the projection of y onto the orthogonal complement space of the span of column in Φ_{I_s}:

$$\begin{aligned} r_s &= y - \Phi_{I_s} x_s \\ &= y - \Phi_{I_s} \Phi^{\dagger}_{I_s} y \ . \\ &= \mathrm{P}^{\perp}_{I_s} y \end{aligned}$$

Then, at the s-th stage of the SmOMP, the following relation can be further obtained:

$$\begin{aligned} \langle \Phi_{I_s}, r_s \rangle &= \langle \Phi_{I_s}, \mathrm{P}^{\perp}_{I_s} y \rangle \\ &= \Phi^T_{I_s} \mathrm{P}^{\perp}_{I_s} y \\ &= (\mathrm{P}^{\perp}_{I_s} \Phi_{I_s})^T y \\ &= [(\mathrm{I} - \mathrm{P}_{I_s}) \Phi_{I_s}]^T y \\ &= (\Phi_{I_s} - \Phi_{I_s} \Phi^{\dagger}_{I_s} \Phi_{I_s})^T y \\ &= 0. \end{aligned}$$

Hence, the residual r_s of the SmOMP is orthogonal to the columns of Φ_{I_s}. Furthermore, indices in I_s can not be reselected in the succeeding iterations. Note that when the iteration loop of the SmOMP is finished, straightforward algebraic operations show that

$$\begin{aligned} \hat{x}_s &= \mathrm{P}_{I_s} y \\ &= (\Phi^T_{I_s} \Phi_{I_s})^{-1} \Phi^T_{I_s} y \\ &= (\Phi^T_{I_s} \Phi_{I_s})^{-1} \Phi^T_{I_s} \Phi_{\Lambda} x_{\Lambda} \\ &= (\Phi^T_{I_s} \Phi_{I_s})^{-1} \Phi^T_{I_s} (\Phi_{I_s} x_{I_s} - \Phi_{I_s - \Lambda} x_{I_s - \Lambda}) \\ &= x_{I_s}. \end{aligned}$$

Since the final support set I_s of the estimation $\hat{x}_s$ contains indices not in Λ, the vector x_0 with entries outside of I_s is the zero. Finally, $y = \Phi_{I_s} x_{I_s} = \Phi_{I_s} x_0$. Note that there is no nontrivial relation $0 = \Phi_{I_s}(x_{I_s} - x_0)$ where $x_{I_s} - x_0$ is a column vector compatible with Φ_{I_s}. Therefore, it is apparent that

$$\hat{x}_s = x_0.$$

This completes the proof of Theorem 1.
